# Neural network models for influenza forecasting with associated uncertainty using Web search activity trends

**DOI:** 10.1371/journal.pcbi.1011392

**Published:** 2023-08-28

**Authors:** Michael Morris, Peter Hayes, Ingemar J. Cox, Vasileios Lampos

**Affiliations:** 1 University College London, Centre for Artificial Intelligence, Department of Computer Science, London, United Kingdom; 2 University of Copenhagen, Department of Computer Science, Copenhagen, Denmark; Washington State University, UNITED STATES

## Abstract

Influenza affects millions of people every year. It causes a considerable amount of medical visits and hospitalisations as well as hundreds of thousands of deaths. Forecasting influenza prevalence with good accuracy can significantly help public health agencies to timely react to seasonal or novel strain epidemics. Although significant progress has been made, influenza forecasting remains a challenging modelling task. In this paper, we propose a methodological framework that improves over the state-of-the-art forecasting accuracy of influenza-like illness (ILI) rates in the United States. We achieve this by using Web search activity time series in conjunction with historical ILI rates as observations for training neural network (NN) architectures. The proposed models incorporate Bayesian layers to produce associated uncertainty intervals to their forecast estimates, positioning themselves as legitimate complementary solutions to more conventional approaches. The best performing NN, referred to as the iterative recurrent neural network (IRNN) architecture, reduces mean absolute error by 10.3% and improves skill by 17.1% on average in nowcasting and forecasting tasks across 4 consecutive flu seasons.

## Author summary

Modelling the prevalence of an infectious disease enables public health organisations to prepare for and minimise its impact. However, traditional disease indicators are often quite restrictive as they provide information with a significant delay. Recent research efforts have provided evidence of the value of alternative information sources such as Web search activity trends. Our work incorporates this information into influenza forecasting models to achieve state-of-the-art accuracy. In addition, the proposed Bayesian neural network architectures also provide associated uncertainty estimates for the forecasts, positioning our methodology as a practical complementary tool for disease surveillance and policy making.

## Introduction

Forecasting the spread of infectious diseases can inform public health policy decisions. The potential impact of forecasting was highlighted during the COVID-19 pandemic where disease incidence and mortality projections led governments to initiate lockdowns [1–3]. There continues to be a considerable interest in forecasting, particularly of influenza [4–21]. According to the World Health Organization, influenza remains a strong candidate for a pandemic and is typically responsible for 290, 000 to 650, 000 deaths worldwide each year. Forecasting its prevalence allows policy makers to, for example, identify when to recommend the prescription of anti-viral drugs [22]. The United States of America (US), like many other countries, has a syndromic surveillance network, coordinated by the Centres for Disease Control and Prevention (CDC), that tracks the rate of influenza-like illness (ILI). Models for influenza forecasting considered by the CDC [23–25] incorporate associated uncertainty estimates as an essential component for deployment within a decision support system. After all, an unexpected forecast with high uncertainty is very different to an unexpected forecast with low uncertainty. The latter might trigger a public health intervention, while the former might be ignored.

Neural networks (NNs) have been shown to be competitive with the state-of-the-art in forecasting tasks [26–28]. However, their application to epidemic forecasting has been limited [29], in part, because estimating uncertainty with NNs can be challenging. The uncertainty in a forecast produced by a machine learning model is attributed to two sources [30]. Data or aleatoric uncertainty is inherent in the data, such as measurement noise. Model or epistemic uncertainty deals with confidence in the model’s parameters [31]. In our work, we use Bayesian neural networks (BNNs) to estimate the model uncertainty. We place a distribution over the NN’s parameters (weights), and sample from that distribution to create model parameter instances. The variation in outputs across the model instances is used to derive the model uncertainty. Data uncertainty is estimated by outputting the parameters of the data distribution, that is a mean prediction and its variance, rather than a single point estimate. We then combine the two uncertainty mechanisms.

At its simplest, a forecast may be based on its own historical values. However, in many cases, better accuracy is achieved by incorporating additional exogenous data. Prior work has demonstrated that the current influenza rate can be accurately estimated (nowcasted) from a variety of different data sources, including social media posts and Web search activity [32–36]. These streams of information have very low latency. Their daily frequency can theoretically be determined with a delay of about 24 hours, i.e. right after the completion of a day. In contrast, syndromic surveillance networks for influenza (including CDC’s) report ILI rates with latencies of about two weeks, i.e. the ILI rate today is not known until two weeks later. Hence metadata about online user activity, when used appropriately, can facilitate more timely disease rate inferences, which perhaps is the most important factor for incorporating this exogenous information into conventional epidemiological approaches. Our proposed NN architectures for disease rate forecasting can efficiently and effectively incorporate frequency time series of Web search queries. We use the daily frequency of a variety of search terms (keywords or phrases) related to ILI. These include symptoms, remedies, general advice seeking, and other relevant categories (S1 Table).

However, the different latencies of health reporting and Web search activity can introduce a level of confusion with respect to model configuration and evaluation. Generally in forecasting we have a set of observed data points (samples) up to and including time (day) *t*_0_, and aim to predict a future value (here a disease rate) at time *t* > *t*_0_. Due to the different data reporting latencies, we can obtain historical ILI rates up to time *t*_0_ and exogenous data up to *t*_0_ + *δ*, where *δ* is typically 14 days. When we refer to the number of days ahead to be forecast, i.e. the forecast horizon denoted by *γ*, we need to specify from what time. For that purpose, we can either use *t*_0_ (time point of the last available ILI rate) or *t*_0_ + *δ* (time point of the most recent exogenous information). Here, we adopt the convention from prior literature and use *t*_0_. As such, a 7 days ahead forecast, i.e. for day *t* = *t*_0_ + 7, with a latency of *δ* = 14 days may actually use exogenous data that is available after the forecast horizon (days *t*_0_ + 8, …, *t*_0_ + *δ*). This is a curious situation, but we note that it is accepted practice within the ILI forecasting community (often referred to as hindcasting), and hence we have chosen to include these results. Obviously, for forecast horizons greater or equal to *δ*, no “future” exogenous data is available, which makes the outcomes of these experiments more relevant in practical terms.

We propose and evaluate the performance of three NN architectures, namely a simple feedforward network (FF) and two forms of recurrent neural networks (denoted SRNN and IRNN; see Methods) all of which incorporate the frequency time series of various Web search terms as exogenous variables, and provide uncertainty estimates by deploying BNN layers and inference techniques. The forecast targets are US national ILI rates as published by the CDC. Evaluation is performed for the four flu seasons from 2015/16 to 2018/19 (both inclusive). For the overall best performing NN model, IRNN, we also confirm that the incorporation of exogenous data significantly improves performance. The best performing networks for each forecasting horizon, SRNN when *γ* = 7 and IRNN otherwise, are then compared with Dante [21], a state-of-the-art conventional ILI forecasting model. Our experiments show that the proposed NN architectures that incorporate Web search activity can significantly reduce forecasting error and provide significantly earlier insights about emerging ILI trends.

## Results

We first provide a comparative performance analysis of the NN-based models. Then, we compare with the established state-of-the-art in ILI forecasting. Details about the models, training, and evaluation can be found in the Methods section.

### Forecasting performance of NNs

We investigate the performance of three Bayesian NN architectures, a feedforward network (FF), a simple recurrent NN optimised for a single forecast horizon (SRNN), and an iterative RNN which feeds back daily forecasts to itself up to and including the horizon window (IRNN). We forecast the national level weighted ILI rate (wILI; see definition in the Methods section) in the US over four flu seasons, namely 2015/16 to 2018/19 from late October until June (exact dates are provided in S2 Table and corresponding ILI rates are displayed in S2 Fig). We evaluate our models for four forecast horizons *γ* = 7, 14, 21, and 28 days ahead from the last available ILI rate. The input to all NNs is both past ILI rates as well as time series of Web search query frequencies. In addition to that, for a more complete comparison, we also report performance results for the best performing NN, IRNN, after excluding Web search activity data. We deploy six metrics to compare estimated forecasts to reported ILI rates (ground truth). Mean absolute error (MAE) and bivariate correlation (*r*) compare forecasts without considering the associated uncertainty. Negative log likelihood (NLL), continuous ranked probability score (CRPS) a generalisation of MAE, and Skill weight the error by its corresponding uncertainty. The (dis)advantages of the various weightings are discussed in S1 Appendix. For NLL, CRPS, and MAE a lower score is better, while for *r* and Skill higher scores are better. When average metrics are calculated across several seasons or forecast horizons the arithmetic mean is used for all metrics besides Skill, where the geometric mean is used [15].

Table 1 enumerates the performance metrics for the three NNs in each flu season and forecast horizon. The IRNN performs best for all forecast horizons, except for *γ* = 7 days ahead where SRNN is the best performing model. As we detail in Methods, this is something expected given the model design. IRNN, contrary to SRNN and FF, is not using future query frequencies (from the 7-days following the target forecast date) for the hindcasting task (*γ* = 7). Interestingly, we also observe that the performance of IRNN does not change for *γ* = 7 and 14, something that can probably be explained by a model behaviour that gives significantly more importance to the more recent inputs (search query frequencies are ahead of the past ILI rates by *δ* = 14 days). IRNN, the most advanced NN that we propose, compared to the next best NN architecture reduces error by 14.87% in terms of MAE, 20% in terms of CRPS, and improves Skill by 32.48%, when averaged across all test seasons and forecasting horizons *γ* = 14, 21, and 28 days. IRNN yields further improvements in the rest of the metrics, although these have a more limited interpretability. The fact that IRNN improves gains between MAE and CRPS (by 4.15 percentage points) means that it is also a better model for the uncertainty bounds compared to FF and SRNN.

**Table 1:** Regression performance metrics for three Bayesian NN models for four forecast horizons (*γ* = 7, 14, 21, and 28 days ahead). Negative log likelihood (NLL), continuous ranked probability score (CRPS) and Skill compare the accuracy weighted by the uncertainty of forecasts. MAE is the mean absolute error, and *r* is the bivariate correlation between forecasts and reported ILI rates. Best results for each metric and forecast horizon are shown in bold. The last three columns are performances averaged over the four test flu seasons (from 2015/16 to 2018/19).

		2015/16			2016/17			2017/18			2018/19			Avg (2015–19)		
*γ*		IRNN	SRNN	FF	IRNN	SRNN	FF	IRNN	SRN	FF	IRNN	SRNN	FF	IRNN	SRNN	FF
7	NLL	0.20	**-0.38**	-0.19	0.38	**-0.29**	-0.06	0.65	**0.07**	0.52	0.32	**-0.49**	0.12	0.39	**-0.27**	0.10
	CRPS	0.18	**0.09**	0.10	0.24	**0.12**	0.14	0.31	**0.17**	0.33	0.21	**0.08**	0.19	0.23	**0.12**	0.19
	Skill	0.73	**0.95**	0.89	0.59	**0.85**	0.78	0.49	**0.77**	0.54	0.62	**0.95**	0.70	0.60	**0.87**	0.72
	MAE	0.26	**0.13**	0.13	0.33	**0.18**	0.20	0.37	**0.25**	0.49	0.28	**0.11**	0.27	0.31	**0.17**	0.27
	*r*	0.85	**0.97**	0.97	0.92	**0.99**	0.99	0.98	**0.99**	0.98	0.93	**0.99**	0.97	0.92	**0.98**	0.98
14	NLL	**0.18**	0.35	0.49	**0.36**	0.53	0.46	**0.64**	3.28	0.94	0.30	**0.27**	0.64	**0.37**	1.11	0.63
	CRPS	**0.18**	0.21	0.20	0.24	0.27	**0.19**	**0.31**	0.72	0.39	**0.21**	0.21	0.27	**0.24**	0.35	0.26
	Skill	**0.73**	0.69	0.61	**0.59**	0.54	0.59	**0.49**	0.11	0.39	0.63	**0.67**	0.52	**0.60**	0.40	0.52
	MAE	0.26	0.29	**0.25**	0.33	0.36	**0.22**	**0.38**	0.88	0.50	**0.28**	0.29	0.40	**0.31**	0.45	0.34
	*r*	0.85	0.85	**0.91**	0.92	0.92	**0.97**	**0.98**	0.88	0.94	0.93	**0.94**	0.91	0.92	0.90	**0.93**
21	NLL	**0.32**	0.85	0.85	**0.63**	0.80	0.88	**0.78**	2.08	1.50	**0.47**	0.88	1.06	**0.55**	1.15	1.07
	CRPS	**0.23**	0.33	0.28	0.31	**0.29**	0.30	**0.42**	0.70	0.61	**0.27**	0.33	0.38	**0.30**	0.41	0.39
	Skill	**0.65**	0.51	0.45	**0.49**	0.46	0.42	**0.43**	0.15	0.24	**0.56**	0.45	0.37	**0.52**	0.36	0.36
	MAE	**0.32**	0.45	0.36	0.42	0.39	**0.35**	**0.56**	0.90	0.79	**0.37**	0.46	0.53	**0.42**	0.55	0.51
	*r*	**0.81**	0.68	0.77	0.85	0.89	**0.93**	**0.94**	0.89	0.86	**0.88**	0.85	0.84	**0.87**	0.83	0.85
28	NLL	**0.53**	1.07	1.07	**0.73**	1.19	1.09	**1.32**	3.82	1.80	**0.54**	0.98	1.27	**0.78**	1.76	1.31
	CRPS	**0.31**	0.40	0.35	**0.35**	0.35	0.38	**0.57**	0.88	0.80	**0.30**	0.36	0.46	**0.38**	0.50	0.50
	Skill	**0.54**	0.41	0.36	**0.45**	0.34	0.35	**0.27**	0.06	0.18	**0.53**	0.40	0.30	**0.43**	0.24	0.29
	MAE	0.45	0.56	**0.40**	0.49	0.44	**0.41**	**0.73**	1.05	1.07	**0.43**	0.49	0.63	**0.53**	0.64	0.63
	*r*	**0.80**	0.55	0.60	0.81	0.89	**0.92**	**0.91**	0.84	0.76	0.85	**0.86**	0.77	**0.84**	0.78	0.76

**Fig 1:**
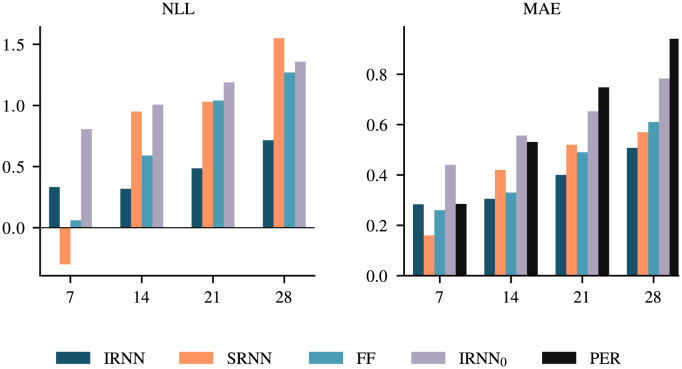
Negative log-likelihood (NLL) and mean absolute error (MAE) for each NN model averaged over all four test flu seasons (2015/16 to 2018/19). Scores for different forecast horizons (*γ*) are shown. Lower values are better. We also provide a comparison with IRNN trained without using any Web search activity data (IRNN_0_), and a simple persistence model (PER). Note that NLL cannot be determined for PER as it does not provide an associated uncertainty. S1 Fig shows the results for all metrics.

Fig 1 provides an alternative visual of the forecasting performance metrics of the different NN models when averaged over the four flu seasons (NLL and MAE are depicted, the rest of the metrics are displayed in S1 Fig). In addition to the three NNs, we also provide performance metrics for an IRNN variant that does not use any search query frequency data (denoted by IRNN_0_) as well as for a simple persistence model (denoted by PER; see S1 Appendix for a definition). IRNN consistently performs better than IRNN_0_, which confirms our hypothesis that Web search activity information provides a significant performance improvement. On the other hand, IRNN_0_ displays competitive performance when compared to SRNN or FF which highlights that IRNN is a more suitable model for handling search query frequency time series. In S4 Table, we have also provided an additional baseline comparison with an elastic net [37] model that, in line with our previous work [34], provides inferior performance (S4 Table and S6 Fig). A fair comparison with Gaussian Processes models [38], that we have also deployed in the past [36, 39], was not practically tractable given the high dimensionality of the task and the relatively large amount of training samples. Finally, the persistence model baseline is always inferior to at least one of the NN models.

**Fig 2:**
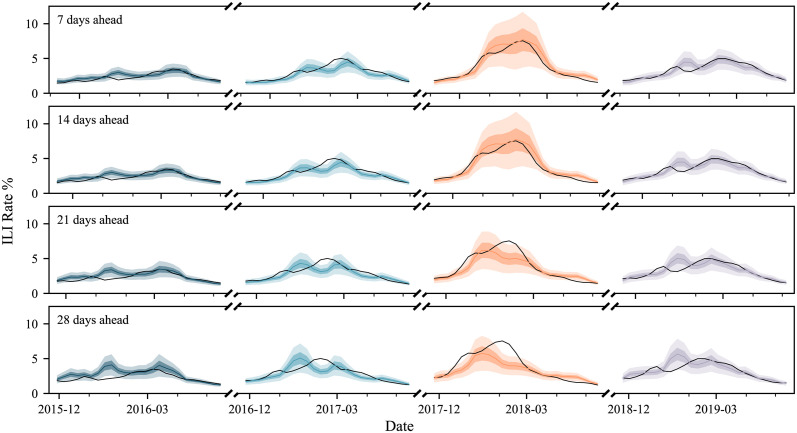
IRNN forecasts for all 4 test seasons (2015/16 to 2018/19) and forecasting horizons (*γ* = 7, 14, 21, and 28). Confidence intervals (uncertainty estimates) are shown at 50% and 90% levels, and are visually distinguished by darker and lighter colour overlays respectively. The influenza-like illness (ILI) rate (ground truth) is shown by the black line.

Forecasts from IRNN in every season and forecast horizon are shown in Fig 2, whereas forecasts from the FF and SRNN architectures are shown in the Supporting Information (S7 and S8 Figs, respectively). The expected decline in accuracy as the forecast horizon increases is visually evident for all models. Interestingly, forecasts from the FF NN follow closely the estimates of a persistence model (i.e. shifted ground truth), and also have quite pronounced uncertainty bounds for *γ* = 21 and 28. SRNN provides smoother but generally flatter forecasts that in principle may capture the underlying ILI trend. However, they quite often underestimate the exact flu rate and are over-confident (tight uncertainty bounds). The IRNN makes more independent forecasts that do not necessarily follow previous trends in recently observed ILI rates. Uncertainty bounds increase slightly with *γ*, albeit we note that this model does not directly differentiate between forecasting horizons. Overall, forecasts from IRNN have a better correspondence to the ILI rate range and provide an early flu onset warning (in at least 3 of the 4 test seasons).

**Fig 3:**
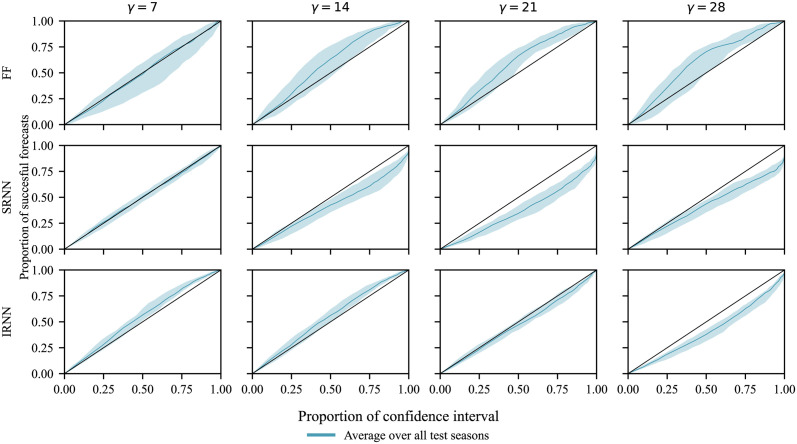
Calibration plots for the forecasts made by the three NN models (FF, SRNN, and IRNN) averaged over the four test periods (2015/16 to 2018/19) and shown for the 4 forecasting horizons (*γ*). The lines show how frequently the ground truth falls within a confidence interval (CI) of the same level. To be more precise, a point (*x*, *y*) denotes that the proportion *y* ∈ [0, 1] of the forecasts when combined with a CI at the *x* × 100% level include the ground truth (successful forecasts). The optimal calibration is shown by the diagonal black line. Points above or below the diagonal indicate an over- or under-estimation of uncertainty, and hence an under- or over-confident model, respectively. The shadows show the upper and lower quartile of the calibration curves when the models are trained multiple times with different initialisation seeds. The plot broken out into separate test periods is shown in the Supporting Information (S11 Fig).

Fig 3 shows the calibration of the confidence intervals (CI) for each of the NNs. The *x*-axis represents the expected frequency that the ground truth data will be present in a specified region of confidence, while the *y*-axis represents the empirical frequency as measured from the test results. Remember that each forecast has an associated uncertainty represented by a Gaussian distribution. For a specified probability, *ρ*, we can determine the confidence region around each forecast such that we expect the ground truth to fall within these regions with probability *ρ*. *ρ* can be computed by *ρ* = cdf(*n*) − cdf(−*n*), where *n* is the number of standard deviations away from the mean, and cdf denotes the cumulative distribution function. For a given probability (on the *x*-axis), we compute the empirical probability for each of the four test seasons. The diagonal line (*y* = *x*) represents perfect calibration, i.e. the expected and empirical probabilities are the same. Points above the diagonal indicate that the uncertainty estimates are too large. Conversely, points below it indicate that the uncertainty estimates are too low. The shadow around the calibration curve shows the variation due to different initialisation seeds over 10 NN training runs (see Methods for further details). Uncertainties produced by the IRNN are closer to the diagonal (i.e. better estimates of uncertainty) for horizon windows greater than 7. Overall, we see that FF is an under-confident model, SRNN an over-confident model, and IRNN generally more balanced, but the error in confidence increases for the largest forecast horizon (*γ* = 28).

### Comparison with state-of-the-art

We compare our best model for each forecasting horizon i.e., SRNN for *γ* = 7 and IRNN for *γ* ≥ 14, to a state-of-the-art ILI rate forecasting model, known as ‘Dante’ [21]. Its original implementation, Dante produces a binned forecast and does not permit comparison based on CRPS or NLL (see S1 Appendix). Therefore, for this analysis we restrict the performance metrics to Skill, MAE, bivariate, and correlation.

To be consistent with prior published literature and conduct a fair comparison, we adopt exactly the same training setup as proposed in the original paper that proposed Dante [21]. However, we would like to make the reader aware of various caveats in this comparison. First, Dante’s national US ILI rate forecasts are based on ILI rates from 63 subnational US geographical regions (50 US states, 10 Health and Human Services regions, the district of Columbia, Puerto Rico, and Guam) as well as ILI rates at the national level. The NNs use only national US ILI rates, augmented with a US national aggregate of Web search activity data. The latter is more recent, i.e. search query frequencies are available until *t*_0_ + *δ* which is after the last observed ILI rate (*t*_0_). To remove this temporal advantage, we do not use Web search activity data generated after *t*_0_ when training models for comparing with Dante. Secondly, Dante is trained using a leave-one flu season-out methodology, training on all other flu seasons (past and future) but the test one. Thus, for example, for the test season 2016/17, Dante will use historical data prior to 2016 and after 2016/17. We do not consider this appropriate as, in practice, a deployed system has no knowledge of future seasons. However, for comparison purposes, we train our models using leave-one flu season-out as well. We note that we were not able to successfully train Dante when restricting training data to exclude future seasons; Dante’s performance was much worse to be considered for a comparison. We emphasise that training on dates after the test season is only done when comparing to Dante. Another caveat is that Dante exploits regional ILI prevalence to come up with a national forecast—this can sometimes provide an earlier warning as outbreaks will first be recorded sub-nationally. Our models are not built this way, and cannot leverage from this information. The final remark is that Dante performs retraining prior to conducting a forecast. Although that is possible for the NN models as well, running complete experiments (across many seasons, different NN architectures, and different initialisation seeds) with retraining every time prior to making a forecast would have taken a considerable amount of time. Hence, NNs make forecasts for an entire flu season without retraining.

Table 2 shows the metrics for the best NN for each forecast horizon *γ*, trained with leave-one flu season-out and with search data from *t* ≤ *t*_0_, and results for Dante taken on identical forecast dates. When averaged over all forecasting tasks, the NNs have 11.93% higher Skill, 4.97% lower MAE, and 5.96% higher correlation than Dante. Dante has a better calibrated uncertainty compared to IRNN, but this can be interpreted by its significantly larger uncertainty estimates that sometimes are over 2 times greater than the ones produced by IRNN (S9 Fig). In general, a better calibrated uncertainty is less important when forecast error metrics indicate an overall inferior performance. The last column (NN_*b*_) of Table 2 provides an expanded comparison (full results are shown in S4 Table) whereby we have enabled training with Web search activity data that maintain their actual latency (*t*_0_ + *δ*). As expected, the performance benefits increase, obtaining 33.52% higher Skill, 14.37% lower MAE, and 8.78% higher correlation compared to Dante. Disabling leave-one flu season-out training on our end also results in a better performance compared to Dante that maintains its knowledge of future flu seasons (see column NN_*a*_ of Table 2).

**Table 2:** Forecasting performance metrics for the best-performing neural network (SRNN for *γ* = 7, IRNN for *γ* ≥ 14) compared with Dante. The NNs are trained using search query frequencies generated only up to the last available ILI rate (the 2-week advantage of using Web search data is removed). We use leave-one flu season-out to train models, similarly to Dante. The best results for this comparison are shown in bold. The very last column (NN_*b*_) presents the average performance results of NNs where the temporal advantage of Web search activity information is maintained (see also S10 Fig that depicts IRNN’s forecasts when leave-one flu season-out is applied). The penultimate column (NN_*a*_) holds results for the same experiment as NN_*b*_ with the addition of disabling leave-one flu season-out training.

Horizon	Metric	2015/16		2016/17		2017/18		2018/19		Avg (2015–19)			
*γ*		Dante	NN	Dante	NN	Dante	NN	Dante	NN	Dante	NN	NN_*a*_	NN_*b*_
7	Skill	0.67	**0.75**	**0.63**	0.53	0.45	**0.53**	**0.62**	0.61	0.59	**0.60**	0.85	0.88
	MAE	**0.22**	0.26	**0.19**	0.35	0.39	**0.38**	**0.21**	0.28	**0.25**	0.32	0.18	0.17
	*r*	**0.88**	0.81	**0.96**	0.91	0.97	**0.98**	**0.97**	0.90	**0.94**	0.90	0.98	0.98
14	Skill	0.54	**0.74**	**0.54**	0.53	0.29	**0.53**	0.52	**0.61**	0.46	**0.59**	0.55	0.59
	MAE	0.38	**0.28**	**0.32**	0.35	0.64	**0.39**	0.33	**0.28**	0.42	**0.33**	0.35	0.34
	*r*	0.64	**0.79**	0.91	**0.91**	0.90	**0.98**	**0.92**	0.90	0.84	**0.89**	0.89	0.89
21	Skill	0.44	**0.64**	**0.48**	0.43	0.21	**0.30**	0.46	**0.52**	0.38	**0.45**	0.47	0.48
	MAE	0.48	**0.37**	**0.38**	0.45	0.86	**0.62**	**0.40**	0.44	0.53	**0.47**	0.48	0.46
	*r*	0.36	**0.67**	**0.87**	0.83	0.82	**0.94**	**0.89**	0.82	0.73	**0.81**	0.81	0.81
28	Skill	0.37	**0.53**	**0.46**	0.38	**0.17**	0.14	0.42	**0.45**	0.33	**0.33**	0.37	0.40
	MAE	0.54	**0.47**	**0.39**	0.50	1.06	**0.85**	**0.45**	0.58	0.61	**0.60**	0.61	0.58
	*r*	0.23	**0.63**	**0.88**	0.79	0.76	**0.92**	**0.86**	0.79	0.68	**0.78**	0.78	0.79

## Discussion

We have demonstrated the ability of neural networks to forecast ILI rates by incorporating exogenous Web search activity data while providing uncertainty estimates. IRNN exhibits superior performance (averaged over all test years) for forecast horizons greater than 7 days, whereas SRNN is superior for the *γ* = 7 days ahead forecast horizon, a prediction task also referred to as hindcasting. As discussed extensively (see Methods and Results), this is expected because when *γ* = 7 days, SRNN is using all the available Web search activity data, which extends 7 days beyond the target forecasting horizon. We have also demonstrated that the proposed forecasting framework can provide very competitive performance that is better than the established state-of-the-art in ILI rate forecasting.

Our experiments highlight the importance of including Web search activity for forecasting ILI rates with or without their expected temporal advantage. This is consistent with previous literature whereby the added value of online user-generated data streams (e.g. Web search, but also social media) has been evaluated [5, 34, 40]. However, our experiments present the most comprehensive analysis to date, assessing performance over 4 consecutive flu seasons, and utilising an open-ended, non-manually curated set of search queries. In addition, we have cross-examined accuracy with a number of different error metrics, including CRPS and NLL that can incorporate the validity of uncertainty estimates. We have seen that adding Web search information not only improves accuracy, but also provides better estimates of confidence (S1 Fig).

By examining ILI seasons in our training and test sets, we can deduce that the 2015/16 test season is the least similar season to previously seen ones (mean bivariate correlation of 0.74), whereas the 2018/19 is the most similar (mean bivariate correlation of 0.81). With that in mind, we observe that in comparison to Dante the NNs that utilise Web search activity perform better when the flu season has a more novel prevalence trajectory (Table 2). As Dante is utilising ILI rates only (including subnational ones), it is expected to be a more focused model on previously seen ILI rate trajectories. In contrast, the search query frequency time series provide an opportunity to capture more complex underlying patterns, and hence seem to be a more informative source during novel flu seasons.

From an epidemiological perspective, accurate forecast estimates might not always be the sole determinant of model superiority. Although, our model performance analysis is comprehensive, and contrary to most of the related literature, provides a clean depiction of seasonal forecasts, it does focus on the accuracy of a forecast and its associated uncertainty. Table 3 attempts to address that partially by offering a few additional comparative insights following aspects of a similar analysis for ILI rate nowcasting models in England [22]. Focusing on the most challenging forecasting horizons (*γ* = 21 and 28 days), we compute the delay in forecasting the peak of the flu season as well as the difference in magnitude between the predicted and the estimated peak ILI rate. We see that Dante is making either very invalid early estimates (e.g. 70 days prior to the actual peak) or otherwise lags by 1 or 2 weeks (i.e. no early warning), whereas the NN models tend to always provide reasonable early warnings of the peak. While there is no definitive winner in estimating the ILI rate peak magnitude, by examining forecasts when the ILI rate was relatively high (above the seasonal mean plus one standard deviation), we observed that Dante’s estimates were significantly worse in terms of MAE and relative MAE (symmetric mean absolute percentage of error). A similar analysis across NN variants is provided in S5 Table highlighting the expected superiority of IRNN.

**Table 3:** Meta-analysis of ILI rate forecasts around the peak of a flu season for Dante, NN (the best NN variant when the temporal advantage of Web search activity data is removed), and NN_*b*_ (same as NN but after reinstating the temporal advantage of Web search activity data). *δ*-p denotes the temporal difference (in days) in forecasting the peak of the flu seasons 2015/16, 2016/17, 2017/18, and 2018/19, respectively. Negative / positive values indicate an earlier / later forecast; averaging *δ*-p across the 4 test flu seasons would remove this information and that is why we enumerate all 4 values. Avg. *δ*-*y*_p_ measures the average magnitude difference in the estimate of the peak of the flu season between a forecasting model and CDC. MAE-p is the MAE when the ILI rate is above the seasonal mean plus one standard deviation. SMAPE-p (%) is the symmetric mean absolute percentage of error for the same time periods. Outcomes that yield an unfavourable interpretation for the underlying forecasting model are provided in bold. Detailed outcomes for all NNs are shown in S5 Table.

Horizon	*γ* = 21		
Metric	Dante	NN	NN_*b*_
*δ*-p (days)	**-70**, 14, 14, 14	-49, 14, -14, -35	-49, 14, -28, -35
Avg. *δ*-*y*_p_	0.99	0.70	0.59
MAE-p	0.84	0.75	0.76
SMAPE-p (%)	20.19	15.57	15.69
Horizon	*γ* = 28		
Metric	Dante	NN	NN_*b*_
*δ*-p (days)	**-70**, 14, 7, 14	-42, -21, -21, -28	-42, -21, -21, -28
Avg. *δ*-*y*_p_	0.67	1.01	0.87
MAE-p	1.09	0.89	0.88
SMAPE-p (%)	**26.24**	17.72	17.57

Existing disease forecasting frameworks are difficult to scale, and incorporating additional features or more training data can result in excessive computational cost. This results in a trade-off between model flexibility and the number of exogenous variables a model can handle effectively [11, 41, 42]. An advantage of neural networks is that they are easy to scale; increasing the amount of training instances often results in better overall performance [43]. Overfitting issues, that become more apparent when working with relatively small data sets, are alleviated to an extent by the deployment of a Bayesian layer which averages over parameter values instead of making single point estimates [44]. A lingering disadvantage, however, is that there is no current consensus on estimating uncertainty with NNs in a principled manner. Our methodological approach, presented in the following section, has attempted to address that by considering two modes of uncertainty (epistemic and aleatoric). In addition, given the relatively restricted amount of samples of training neural networks, our experimental approach provides novel insights for model derivation, training, and hyperparameter validation for similar time series forecasting tasks.

It is equally important to acknowledge the limitations of our methodological approach, and more broadly, of this research task as a whole. We note that the retrospective analysis provided in this paper cannot be the only determinant for model deployment within established syndromic surveillance systems. This would also require real-time assessments during ongoing influenza seasons in collaboration with public health organisations. Furthermore, an ILI consultation rate is not always representative of the true influenza rate in a population. It is a proxy indicator, and as such oftentimes it might be biased [45, 46]. Therefore, any model that is trained and evaluated based on these rates is inherently limited by this property. An additional factor that could arguably yield misleading inferences is the co-existence of COVID-19 and influenza, given their similar symptom profiles. Although this is outside the remit of this paper, early results from our ILI models for England during the 2022/23 flu season have showcased that ILI rates can be accurately estimated during COVID-19 outbreaks [47]. From a methodological perspective, we note that our approach in estimating uncertainty can be improved—IRNN, the best performing NN, is currently not explicitly aware of the actual forecasting horizon (*γ*) when conducting a prediction (see Methods). Addressing this in an appropriate way will most likely result in better calibrated uncertainty estimates. From an empirical evaluation perspective, our experiments have been conducted on the US at a national level. Hence, although we expect that these results will generalise sub- and inter-nationally, we have no evidence of this, apart from the fact that past research on similar types of models has shown promise in various different US subregions or countries [36, 39, 48–50]. Finally, the application presented in this paper relies on the existence of Web search activity data. Access to this data is not assured as it both depends on sufficient Internet usage rates as well as on the willingness of private corporations to provide this information for research and epidemiological modelling. Nonetheless, the presented forecasting models do provide a general machine learning approach applicable to different input (e.g. social media activity, body sensors) and output streams of information (e.g. different disease indicators).

## Methods

We first provide our ethics statement, then describe the data sets used, introduce the neural network architectures we have deployed, and finally detail how training and validation was performed.

### Ethics

The project is using publicly available aggregate influenza-like illness rates obtained by the website of CDC. It also uses aggregate (at the national level) search query frequencies obtained by a private Google Health Trends API. Since we only access aggregated data, this project has been exempted from an Ethics review by the department of Computer Science at University College London.

#### Data sets

#### Influenza-like illness rates

CDC defines ILI as fever (temperature of 37.8° or greater) and cough and/or sore throat without a known cause besides influenza. The prevalence of ILI is monitored through several surveillance efforts including the Outpatient Influenza-like-Illness Surveillance Network (ILINet) which collects weekly state level ILI rates from over 2, 000 healthcare providers from all states. The state level ILI rates are weighted by population size to report the wILI at different geographic levels [51]. Our models use weekly wILI rates for the flu seasons 2004/05 to 2018/19 inclusive, obtained from gis.cdc.gov/grasp/fluview/fluportaldashboard.html. Note that this data is not final, i.e. it can be revised by the CDC. To ensure reproducibility of our results, a copy of all the ILI data used can be found at our Github repository github.com/M-Morris-95/Forecasting-Influenza-Using-Neural-Networks-with-Uncertainty. A week in the CDC data represents a 7-day period that starts on a Sunday and ends on a Saturday. We assume the weekly ILI rate is representative of Wednesday (middle day) and use cubic interpolation (interpolate.interp1d from Python’s SciPy library) to generate daily ILI rates. This not only increases the number of samples (7-fold), but also provides an aligned time series with the daily temporal resolution of the Web search activity data. The deployment of a cubic as opposed to a linear interpolation to generate daily ILI rates resulted in slightly better forecasting accuracy on the test sets. We hypothesise that this is because of the increased level of smoothness (see S5 Fig), but also note that we have not fully assessed this data manipulation choice. For training the Dante forecasting model, we have also obtained regional wILI rates for the 53 US states / locations and the 10 US Health and Human Services regions. These were downloaded from the CDC for the same period above and are also available on our Github repository.

#### Search query frequency time series

Search query frequencies for the US are obtained from the Google Health Trends API similarly to other studies [36, 52]. A frequency represents the fraction of searches for a certain term or set of terms divided by the total amount of searches (for any term) for a day and a certain location. We initially downloaded the daily search frequencies of a predetermined pool of 20, 856 unique health-related search queries for the period from March 2004 to May 2019 inclusive for the US. Query frequencies are smoothed using a 7-day moving average, and min-max normalisation is applied to each query’s time series during training (i.e. without using any future data). For a given test season, for each query, *q*, we compute the correlation score *R*_*q*_ with the ILI rate over the five seasons preceding the test season. We also compute a semantic similarity score *S*_*q*_ that measures each query’s similarity to a predefined flu concept as described in Lampos et al. (2017) [36]. Both scores are then normalised between 0 and 1 and a composite score $U_{q}=R_{q}^{2}+S_{q}^{2}$ for each query is calculated. Only the *m* queries with the highest *U*_*q*_ are used, where *m* is a hyperparameter (see “Hyperparameter optimisation”).

### Neural network architectures

The three NN architectures we have deployed are described next. Each NN outputs two values, namely an ILI rate forecast estimate ($\hat{y}$) and an associated data uncertainty ($\hat{\sigma }$). Each architecture also has an additional Bayesian layer where the weights are specified by an associated probability distribution. Multiple models are instantiated, based on sampling from the weight distribution, and the outputs from these model instances are used to estimate the model uncertainty.

#### Feedforward Neural Network (FF)

The FF model has two hidden feedforward neural layers with a ReLU (max(0, *x*)) activation function, and a Bayesian layer (S12 Fig). The input to the network is a window of *τ* + 1 days of ILI rates and *m* search query frequencies. There is an ILI rate collection delay of *δ* days, in that at day *t*_0_ we know (CDC has published) the ILI rate of day *t*_0_ − *δ*. The delay is assumed to be *δ* = 14 days throughout our experiments. Thus, at day *t*_0_, the input to the network consists of ILI rates, $F_{t_{0}-\tau }$ to $F_{t_{0}}$, and search query frequencies, $Q_{t_{0}+\delta -\tau }$ through $Q_{t_{0}-\delta }$. We ignore the temporal structure of the data and use an (*m* + 1) × (*τ* + 1) vector as the input to the neural network. The output of the network is an estimate of the ILI rate and corresponding data uncertainty *γ* days ahead.

#### Simple Recurrent Neural Network (SRNN)

This is a recurrent neural network which observes a time series of ILI rates and search frequencies (S13 Fig). The input to the network is the same as for FF, but without flattening into a vector. We feed the (*m* + 1) × (*τ* + 1) input matrix into a Gated Recurrent Unit (GRU) layer one day at a time. The final output of the GRU is passed to a dense layer with a distribution over its weights.

#### Iterative Recurrent Neural Network (IRNN)

**Fig 4:**
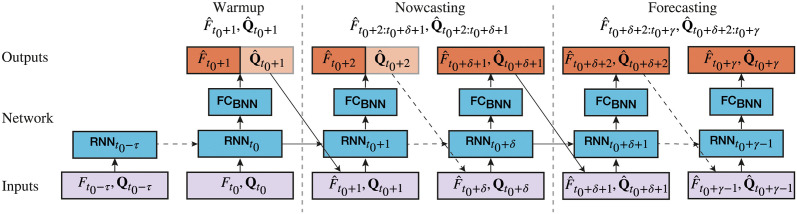
Diagram of the IRNN architecture where for the recurrent layers (RNN) we have used a Gated Recurrent Unit. An ILI rate, *F* ∈ [0, 1], and *m* search query frequencies, $Q\in R_{\geq 0}^{m}$, beginning from time point (day) *t*_0_ − *τ* are fed into the network a day at a time. *τ* denotes the window size of past observations that we consider (*τ* + 1 = 56 days). The reporting delay of the ILI rates means that when an ILI rates are available up to day *t*_0_, search query frequencies are available up to day *t*_0_ + *δ*, where *δ* = 14 days in our experiments. Dashed arrow lines denote that the model is called for multiple time-steps (where a time step is a day). For days *t*_0_ − *τ* to *t*_0_, IRNN enters a warm-up phase where it sets the hidden states in the RNN layer without making any predictions. For days *t*_0_ to *t*_0_ + *δ*, we can observe search query frequencies, but we cannot observe ILI rates. At this stage, IRNN performs nowcasting with respect to input **Q**. During nowcasting the estimated ILI rate ${\hat{F}}_{t}$ is combined with the true search frequencies **Q**_*t*_ use as the input for the next time step. The query search frequency estimates which are not used (as they are known to us) are shown by a faded box. For days *t*_0_ + *δ* + 1 to *t*_0_ + *γ*, where *γ* denotes the forecasting horizon, IRNN conducts pure forecasting as neither search query frequencies nor ILI rates are known for that period. Forecasted values for both of them are used as inputs for subsequent time steps. The full sequence of both predicted ILI rates and search query frequencies is used in the training loss.

This is a recurrent neural network which makes forecasts of the ILI rate and search frequencies one day at a time. It bases forecasts on its own previous forecasts. IRNN comprises a recurrent GRU layer and a feedforward Bayesian layer as shown in Fig 4. We have also described how model training works with pseudocode in the Supporting Information (S14 Fig). Given its special structure, IRNN does not incorporate future (for a period of 7 days after the target forecast) search query frequencies when *γ* = 7. Hence, for both *γ* = 7 and 14, the only minor difference may be due to the more recent past ILI rate inputs. As a result, the difference in performance between *γ* = 7 and 14 is expected to be minor given that search query frequencies are always the more recent information source (as opposed to past ILI rates). This is also empirically confirmed by our experiments (see Table 1). A caveat of the current formulation of IRNN is that the model is agnostic of the actual forecast horizon and hence its uncertainty might be underestimated for larger forecasting horizons.

### Uncertainty estimation using a Bayesian NN layer

We first describe the data uncertainty, then model uncertainty, and finally how the two uncertainties are combined.

#### Data uncertainty

Data or aleatoric uncertainty is caused by noisy observations. It can be further divided into homoscedastic (constant for all inputs) and heteroscedastic (dependent on the input) uncertainty [53]. We estimate heteroscedastic uncertainty in the output layer of the neural network by approximating the parameters of a Gaussian distribution [54]

$$\begin{array}{c} f_{\Phi }\left(x\right)\;=N(\hat{y},\hat{\sigma })=N(a_{1},\text{softplus}\left(a_{2}\right))\;, \end{array}$$ (1)

where *a*_1_ and *a*_2_ are the outputs of the neural network. Softplus is defined by

$$\begin{array}{c} \hat{\sigma }=\frac{1}{s}\;\text{ln}\left(1+e^{c+a_{2}}\right)\;, \end{array}$$ (2)

where *s* > 0 is a scaling factor, and *c* = ln (*e*^*x*^ − 1) is an offset which makes the output equal to 1 when *a*_2_ = 0. All hyperparameters (insofar *s* and *c*) are jointly optimised using Bayesian optimisation (see “Hyperparameter optimisation”). The network is trained by minimising NLL given by

$$\begin{array}{c} \text{NLL}\left(y,\hat{y},\hat{\sigma }\right)=\frac{1}{T}\sum_{t=1}^{T}(\frac{1}{2{\hat{\sigma }}_{t}^{2}}{\left(y_{t}-{\hat{y}}_{t}\right)}^{2}+\frac{1}{2}\;\text{log}\;(2\pi {\hat{\sigma }}_{t}^{2}))\;, \end{array}$$ (3)

where **y** = {*y*_1_, …, *y*_*T*_} is a series of *T* flu rates (ground truth), $\hat{y}=\left\{{\hat{y}}_{1},\ldots ,{\hat{y}}_{T}\right\}$ is a series of *T* forecasts, and $\hat{\sigma }=\left\{{\hat{\sigma }}_{1},\ldots ,{\hat{\sigma }}_{T}\right\}$ is a series of associated standard deviations (data uncertainty). The first component of Eq 3 contains a residual term equivalent to the mean squared error and an uncertainty normalisation term. The second component prevents the model from predicting an infinitely large $\hat{\sigma }$. Minimizing the NLL allows us to train an NN despite not having ground truth estimates of the data uncertainty.

#### Model uncertainty

Model or epistemic uncertainty is inherent in the parameters of the model. It is caused by having insufficient data to exactly set the model’s parameters. To estimate model uncertainty, we deploy a BNN trained using variational inference [55]. BNNs have a distribution over their parameters. For our purposes, we restrict the Bayesian component to the last layer of a network, while preceding layers use deterministic weights and biases. We do this as we found it to be more stable and faster compared to training using a distribution over all the parameters of the model [56]. We use a Gaussian prior with a diagonal covariance matrix $p\left(\Phi \right)=N\left(0,\sigma_{p}I\right)$, where *σ*_*p*_ ∈ [0.0001, 0.1] is a hyperparameter. The form of the distributions is an implementation choice. We specify the posterior distribution to the same form (Gaussian with diagonal covariance) as the prior, such that we have a conjugate prior [57]. The mean *μ*_*w*_ and standard deviation *σ*_*w*_ of each weight in the posterior are learned. ***θ*** holds all the layer’s parameters that are updated during training; the means in the posterior are half of its parameters and the softplus of the standard deviations constitutes the other half. Instances of the model are created by sampling the weights in the Bayesian layer. For each instantiation of the model, a forecast and associated data uncertainty are determined. The variation in the forecast across the model instances provides an estimate of the model uncertainty.

#### Combining data and model uncertainty

Each time that we sample from the posterior distribution of the model’s parameters (which in Experiments will be denoted by *q*_***θ***_(**Φ**)) we create a model instance, *k*, that forecasts a mean ${\hat{y}}_{k}^{\prime }$ and standard deviation ${\hat{\sigma }}_{k}^{\prime }$. After drawing *K* samples, the predictions are combined to create a single distribution. The mean $\hat{y}$ is the mean of the *K* forecasts, i.e.

$$\begin{array}{c} \hat{y}=\frac{1}{K}\sum_{k=1}^{K}{\hat{y}}_{k}^{\prime }\;, \end{array}$$ (4)

and the variance is given by [31]

$$\begin{array}{c} {\hat{\sigma }}^{2}\approx \frac{1}{K}\sum_{\kappa =1}^{K}{\hat{y}}_{\kappa }^{\prime 2}-{\left(\frac{1}{K}\sum_{\kappa =1}^{K}{\hat{y}}_{\kappa }^{\prime }\right)}^{2}+\frac{1}{K}\sum_{\kappa =1}^{K}{\hat{\sigma }}_{\kappa }^{\prime 2}\;. \end{array}$$ (5)

In Eq 5, the first two sum terms define the variance of the means, i.e. the model uncertainty. The third term is the mean of the variances, i.e. the data uncertainty.

### Experiments

We first introduce the training setup for a BNN, and the variations which are used for the different architectures, then we discuss hyperparameter optimisation, and finally how the evaluation is performed in our experiments.

#### Training a BNN

Bayesian inference is used to learn the posterior distribution *p*(**Φ**|**D**) over the model parameters **Φ**, where **D** is the training data containing inputs and targets. In theory, the posterior can be derived by using Bayes rule, i.e.

$$\begin{array}{c} p\left(\Phi |D\right)=\frac{p(D|\Phi )p(\Phi )}{p(D)}\;, \end{array}$$ (6)

where *p*(**Φ**) is a prior distribution which expresses prior belief about the parameter distribution before training data is observed. However, we cannot obtain an exact estimate of the posterior due to the integral *p*(**D**) = ∫_**Φ**_ *p*(**D**, **Φ**′)*d***Φ**′, which is unavailable in closed form and requires exponential time to compute [55]. We instead use variational inference, replacing Eq 6 with an optimisation task. The posterior *p*(**Φ**|**D**) is constrained to a family of distributions F [55], and instead of finding the exact posterior, a variational distribution *q*_***θ***_(**Φ**) is used. ***θ*** is used to describe the distribution [58] e.g., the mean and variance for a Gaussian. The goal of training becomes to learn ***θ*** by minimising the Kullback-Leibler divergence (*D*_KL_) to the true posterior *p*(**Φ**|**D**) as in

$$\begin{array}{c} \underset{\theta }{\text{argmin}}\;D_{\text{KL}}\;[q_{\theta }\left(\Phi \right)\left|\right|p\left(\Phi |D\right)]\;\;\text{such}\;\text{that}\;\;q_{\theta }\left(\Phi \right)\in F\;. \end{array}$$ (7)

The KL divergence represents the average number of bits required to encode a sample from *p*(**Φ**|**D**) using *q*_***θ***_(**Φ**). To compute the KL divergence, *p*(**D**) is required. However, we can instead maximise the evidence-lower-bound (ELBO) given by

$$\begin{array}{c} \text{ELBO}\left(\theta \right)=E\;[\text{log}\;(p(D|\Phi ))]-D_{\text{KL}}\;[q_{\theta }\left(\Phi \right)\left|\right|p\left(\Phi \right)]\;, \end{array}$$ (8)

which is equivalent to Eq 7 up to a constant and is tractable. The first component of Eq 8 is computed using Eq 3, and measures how well the model fits the training data. The second component of Eq 8 is the KL divergence between the posterior and prior distribution. The KL divergence term behaves similarly to a regulariser and encourages the model to choose a simple *q*_***θ***_(**Φ**). When the ELBO is computed using minibatch gradient descent, the gradients are averaged over each minibatch. Therefore, to compute the ELBO the KL divergence term is weighted by the total number of minibatches during training [59]. We also use an additional KL annealing term *ξ*, which limits the regularisation effect of the KL term. For the FF and SRNN models this is equal to 1/*M* where *M* is the number of minibatches multiplied by *KL*_*w*_ [60]. For the IRNN model, *M* is the number of minibatches multiplied by *γ* = 28 and *KL*_*w*_. This is because the model makes 28 outputs, and each requires calling the dense Bayesian layer. The constant, *KL*_*w*_, is chosen by hyperparameter optimisation. Thus, Eq 8 becomes

$$\begin{array}{c} \text{ELBO}\left(\theta \right)=E\;[\text{log}\;(p(D|\Phi ))]-\xi D_{\text{KL}}[q_{\theta }\left(\Phi \right)\left|\right|p\left(\Phi \right)]\;. \end{array}$$ (9)

When training the FF and SRNN models, each training step takes an [(*m* + 1) × (*τ* + 1)]-dimensional input (where *m* denotes the number of search queries and *τ* + 1 denotes the window of days, from *t*_0_ and back, for which query frequencies and ILI rates are used) and produces a forecast estimate ${\hat{y}}_{t_{0}+\gamma }$ containing both a mean and standard deviation for the ILI rate for time (day) *t*_0_ + *γ*. The parameters **Φ** are updated by minimising Eq 9. During each training step one sample is taken from *q*_***θ***_(**Φ**) and used to compute the ELBO. We use backpropagation to compute gradients and update the parameters in both the Bayesian and non-Bayesian layers. The model is retrained for each time horizon *γ*, where *γ* = 7, 14, 21 or 28 days, and for each test period.

The output of the IRNN is a sequence of ILI rates and search frequencies. Although we have search data from *t*_0_ to *t*_0_ + *δ*, we use the full sequence of estimated query frequencies when backpropagating the ELBO (Eq 9) through time. When evaluating the model’s performance we are only concerned with the model’s ILI rate forecasts. The Bayesian layer is called once for each iterative prediction.

#### Hyperparameter optimisation

We use Bayesian hyperparameter optimisation [61] with 5-fold cross validation where each fold is a 365-day period covering a full flu season (see S3 Table and S3 and S4 Figs). We tune the hyperparameters once before the first test period, and keep the same hyperparameters for all subsequent test seasons. For the FF and SRNN the hyperparameters are re-tuned for each of the four forecast horizons. For the IRNN the hyperparameters are tuned once, considering all four forecasting horizons (the average NLL is computed across them). The hyperparameters are the following: the size of the hidden NN layers ∈ [25, 125], the number of queries *m* ∈ [20, 150], the weighting of the KL divergence term in the ELBO loss *KL*_*w*_ ∈ [0.0001, 1.0], the scaling factor of the output’s standard deviation *s* ∈ [1.0, 100], the prior standard deviation *σ*_*p*_ ∈ [0.0001, 0.1], the number of epochs ∈ [10, 100], and the learning rate ∈ [0.0001, 0.01] for training the NNs. After the hyperparameters are tuned we re-train the model using the full training set for the number of epochs chosen. The derived model is then used for forecasting on the test set. Note that hyperparameters are not re-tuned for comparing with Dante (when Web search activity data that are more recent that the last observed ILI rate are removed), which may have disadvantaged our NN models.

#### Inference

When making an estimate with a BNN based on inputs **X**, and with training data **D**, the goal is to compute an output $\hat{y}$ for the entire distribution over **Φ**:

$$\begin{array}{c} p\left(\hat{y}|X,D\right)=\int_{\Phi }p\left({\hat{y}}^{\prime }|X,\Phi^{\prime }\right)p\left(\Phi^{\prime }|D\right)d\Phi^{\prime }\;. \end{array}$$ (10)

In practice $p(\hat{y}|X,D)$ is estimated using Monte Carlo sampling from *p*(**Φ**|**D**) [58]. At prediction time, the posterior distribution over the weights is sampled *K* times, each giving a output $N({\hat{y}}^{\prime },\sigma^{\prime })$. The *K* estimates are combined using Eq 5 which makes an estimate for the combined model and data uncertainty. *K* is chosen by sampling until the final estimate of $\hat{y}$ stabilises. Initially we sample 10 times and produce an estimate using Eq 5. We then run the model a further 10 times and produce a new estimate using the 20 samples. We repeat this process until increasing *K* by 10 does not change the estimated mean by more than 0.1%. Despite averaging over *K* instances of the model, we observed some instability in training the models.

To resolve this each model was trained 10 times with different initialisation seeds, i.e. the seed controlling the initial parameter values of the NN. The mean of the 10 estimates of forecasts and associated variances are our final forecast and variance. We considered alternate methods of combining estimates, such as Eq 5, and averaging the probability density functions. Ultimately we found that averaging the means and variances gave the best final forecasts. Thus, the total number of samples for making a forecast is equal to $\sum_{i=1}^{10}d_{i}\times 10$, where *i* ∈ {1, …, 10} denotes a different seed, and *d*_*i*_ ≥ 20 is the number of samples required for this seed to converge.

To make an estimate with the SRNN and FF models, the inputs are passed through the model’s layers up to the Bayesian layer. The weights in the Bayesian layer are then sampled *K* times, and the estimates from the *K* samples are combined with Eq 5 as discussed in the previous paragraph. Making an estimate with IRNN has three distinct phases: warm-up, nowcasting, and forecasting (S14 Fig). During the warm-up phase the model observes ILI rates and *m* search queries from *t*_0_ − *τ* to *t*_0_. This sets the hidden states of the GRU layer based on all ILI rates and search frequencies from the same days. The output of the GRU is fed into the Bayesian layer (denoted by FC_BNN_ in Fig 4), which estimates the input for the next time step. The Bayesian layer estimates model and data uncertainty, and has 2 × (*m* + 1) units. The first half of the units estimate the means of the query frequencies and ILI rate, the second half of the units estimate the corresponding standard deviations. The estimated ILI rate is a distribution which cannot be directly interpreted by a NN layer. Therefore, a sample from this distribution is combined with the true search query frequencies and fed back into the GRU layer. This is repeated from *t*_0_ to *t*_0_ + *δ* (nowcasting phase). After time *t*_0_ + *δ*, no more search query frequencies are available. The estimated search query frequencies and ILI rates from each time step are fed back into the model to make subsequent forecasts. The process of making daily estimates can be repeated indefinitely, so *γ*, the forecasting horizon, could increased arbitrarily.

#### Evaluation

We evaluate the performance for forecasting horizons *γ* = 7, 14, 21 and 28 days ahead. We choose weekly test dates starting from week 45 and lasting for 25 weeks. We use the 2015/16, 2016/17, 2017/18 and 2018/19 flu seasons to evaluate our model. We did not consider running experiments on data from 2019/20 or 2020/21 as flu prevalence has significantly declined, and ILI rate estimates from CDC became less reliable due to the COVID-19 pandemic. We train models for the period 05/06/2004 until the Wednesday of the 33rd week of the year in which the test flu season starts (around mid-August). We test the models on the period from the Sunday of week 44 until the Saturday of week 23 in the following year. Exact training and test periods are provided in the Supporting Information (S2 Table and S2 Fig). To compare our NN models to Dante we evaluate the model scores on the same test weeks as specified in Reich et al. (2019) [15]. When comparing the best performing NNs to Dante, the training set included all seasons except the test season, i.e. it also included data after the test season (models NN and NN_*b*_ in Table 2). We did not re-tune hyperparameters to account for training on future seasons. As discussed earlier, we do not consider training on data after the test period to be appropriate, but it allows the most direct comparison to the training setup used by Dante. We also report the performance of our best performing NNs when trained using only data prior to the test season (model NN_*a*_ in Table 2).

## Supporting information

S1 Appendix Discussion of metrics and baseline persistence model. Appendix discussing the multibin logarithm score, forecast skill score, continuous ranked probability score, mean absolute error and bivariate correlation metric. As well as the persistence model used the baseline.
(PDF)

S1 Fig Average metrics for all models. Negative log-likelihood (NLL), continuous ranked probability score (CRPS), Skill, mean absolute error (MAE), and bivariate correlation for each NN model averaged over all four test flu seasons (2015/16 to 2018/19). Scores for different forecast horizons (*γ*) are shown. We also provide a comparison with IRNN trained without using any Web search activity data (IRNN_0_) and a simple persistence model (PER) wherever applicable. This figure is a supplement to Fig 1 from the main manuscript.
(EPS)

S2 Fig ILI rates for training and testing. National US ILI rates (as reported by the CDC) for the training (black) and testing (red) periods for each of the 4 test folds that we used in our experiments.
(EPS)

S3 Fig Train and validation ILI rates. National US ILI rates (as reported by the CDC) that we used for determining the hyperparameters of the NNs. We have denoted the 5 training and validation periods with black and red colours respectively.
(EPS)

S4 Fig Validation set diagram. Validation set diagram. Hyperparameters are validated using 5-fold cross validation, where the validation periods are the last five available seasons before the test period. Error (NLL) is averaged over these validation periods. After hyperparameter optimisation, the full training set is used.
(EPS)

S5 Fig Cubic interpolation example. Cubic interpolation of weekly ILI rates (as reported by the CDC for US) to produce pseudo-daily ones. Although cubic interpolation differs slightly from linear interpolation (straight line), it does not distort the weekly signal significantly and produces a more smoothed trend.
(EPS)

S6 Fig Elastic-Net forecasts. Elastic-Net forecasts for all 4 test seasons (2015/16 to 2018/19) and forecasting horizons (*γ* = 7, 14, 21, and 28). The influenza-like illness (ILI) rate (ground truth) is shown by the black line.
(EPS)

S7 Fig FF Forecasts. FF forecasts for all 4 test seasons (2015/16 to 2018/19) and forecasting horizons (*γ* = 7, 14, 21, and 28). Confidence intervals (uncertainty estimates) are shown at 50% and 90% levels, and are visually distinguished by darker and lighter colour overlays respectively. The influenza-like illness (ILI) rate (ground truth) is shown by the black line. The flu seasons are shown in different colours which correspond with the calibration plots on the right. The calibration lines show how frequently the ground truth falls within a confidence interval (CI) of the same level. To be more precise, a point (*x*, *y*) denotes that the proportion *y* ∈ [0, 1] of the forecasts when combined with a CI at the *x* × 100% level include the ground truth (successful forecasts). The optimal calibration is shown by the diagonal black line. Points above or below the diagonal indicate an over- or under-estimation of uncertainty, and hence an under- or over-confident model, respectively. The shadows show the upper and lower quartile of the calibration curves when the models are trained multiple times with different initialisation seeds.
(EPS)

S8 Fig SRNN Forecasts. SRNN forecasts for all 4 test seasons (2015/16 to 2018/19) and forecasting horizons (*γ* = 7, 14, 21, and 28). Confidence intervals (uncertainty estimates) are shown at 50% and 90% levels, and are visually distinguished by darker and lighter colour overlays respectively. The influenza-like illness (ILI) rate (ground truth) is shown by the black line. The flu seasons are shown in different colours which correspond with the calibration plots on the right. The calibration lines show how frequently the ground truth falls within a confidence interval (CI) of the same level. To be more precise, a point (*x*, *y*) denotes that the proportion *y* ∈ [0, 1] of the forecasts when combined with a CI at the *x* × 100% level include the ground truth (successful forecasts). The optimal calibration is shown by the diagonal black line. Points above or below the diagonal indicate an over- or under-estimation of uncertainty, and hence an under- or over-confident model, respectively. The shadows show the upper and lower quartile of the calibration curves when the models are trained multiple times with different initialisation seeds.
(EPS)

S9 Fig Dante Forecasts. Dante forecasts for all 4 test seasons (2015/16 to 2018/19) and forecasting horizons (*γ* = 7, 14, 21, and 28). Confidence intervals (uncertainty estimates) are shown at 50% and 90% levels, and are visually distinguished by darker and lighter colour overlays respectively. The influenza-like illness (ILI) rate (ground truth) is shown by the black line. The flu seasons are shown in different colours which correspond with the calibration plots on the right. The calibration lines show how frequently the ground truth falls within a confidence interval (CI) of the same level. To be more precise, a point (*x*, *y*) denotes that the proportion *y* ∈ [0, 1] of the forecasts when combined with a CI at the *x* × 100% level include the ground truth (successful forecasts). The optimal calibration is shown by the diagonal black line. Points above or below the diagonal indicate an over- or under-estimation of uncertainty, and hence an under- or over-confident model, respectively.
(EPS)

S10 Fig IRNN Forecasts. IRNN forecasts with leave-one flu season-out and using all available Web search data for all 4 test seasons (2015/16 to 2018/19) and forecasting horizons (*γ* = 7, 14, 21, and 28). Confidence intervals (uncertainty estimates) are shown at 50% and 90% levels, and are visually distinguished by darker and lighter colour overlays respectively. The influenza-like illness (ILI) rate (ground truth) is shown by the black line. The flu seasons are shown in different colours which correspond with the calibration plots on the right. The calibration lines show how frequently the ground truth falls within a confidence interval (CI) of the same level. To be more precise, a point (*x*, *y*) denotes that the proportion *y* ∈ [0, 1] of the forecasts when combined with a CI at the *x* × 100% level include the ground truth (successful forecasts). The optimal calibration is shown by the diagonal black line. Points above or below the diagonal indicate an over- or under-estimation of uncertainty, and hence an under- or over-confident model, respectively. The shadows show the upper and lower quartile of the calibration curves when the models are trained multiple times with different initialisation seeds.
(EPS)

S11 Fig Detailed calibration plots for neural network models. Calibration plots for the forecasts made by the three NN models (FF, SRNN, and IRNN) for each of the four test periods (2015/16 to 2018/19) and forecasting horizons (*γ*). The lines show how frequently the ground truth falls within a confidence interval (CI) of the same level. To be more precise, a point (*x*, *y*) denotes that the proportion *y* ∈ [0, 1] of the forecasts when combined with a CI at the *x* × 100% level include the ground truth (successful forecasts). The optimal calibration is shown by the diagonal black line. Points above or below the diagonal indicate an over- or under-estimation of uncertainty, and hence an under- or over-confident model, respectively. The shadows show the upper and lower quartile of the calibration curves when the models are trained multiple times with different initialisation seeds.
(EPS)

S12 Fig Diagram of FF architecture. Diagram of the feedforward (FF) NN architecture with dimensions of parameter matrices shown. *L*_1_ and *L*_2_ denote the number of units in fully connected layers FC_1_ and FC_2_, respectively. FC_BNN_ denotes a fully connected Bayesian layer with a distribution over its weights.
(EPS)

S13 Fig Diagram of SRNN architecture. Diagram of the Simple RNN (SRNN) architecture. GRU (Gated Recurrent Unit) is a recurrent layer, and FC denotes a fully connected dense layer. FC_BNN_ denotes a fully connected Bayesian layer with a distribution over its weights. Note that the query frequencies (*Q*) and the ILI rates (*F*) are temporally misaligned by *δ* days.
(EPS)

S14 Fig IRNN pseudocode. Pseudocode describing how the IRNN model makes one sequence of forecasts up to *γ* days ahead.
(EPS)

S1 Table Example Web search queries. A demonstration of manually curated topics based on the Web search queries used in forecasting models trained for and tested on the 2015/16 flu season. Please note that we do not use query topics in our forecasting models.
(XLSX)

S2 Table Rain and test intervals. The training and testing date intervals (all inclusive) for the four flu seasons used to evaluate forecasting methods in our experiments. Dates given are the days from which forecasts are made.
(XLSX)

S3 Table Train and validation intervals. The training and validation date intervals of the 5 validation folds. These are used to validate and determine the hyperparameter values of the NNs in our experiments.
(XLSX)

S4 Table Performance metrics for best neural network models compared with Dante. Forecasting performance metrics for the best-performing neural network (SRNN for *γ* = 7, IRNN for *γ* ≥ 14) compared with Dante (Dte) and Elastic Net (Eln). The NNs are trained using search query frequencies generated only up to the last available ILI rate (the 2-week advantage of using Web search data is removed). We use leave-one flu season-out to train models, similarly to Dante. The best results for this comparison are shown in bold. NN_*b*_ denotes results where the temporal advantage of Web search activity information is maintained (compared to NN). NN_*a*_ holds results for the same experiment as NN_*b*_ with the addition of disabling leave-one flu season-out training, i.e. training does not include data after the test year. Eln uses the same data sets (inputs, targets) as the NNs. Therefore, it is trained using look ahead and without leave-one flu season-out. Eln does not estimate uncertainty and hence, the Skill metric is not available (empty cell). This Table supplements Table 2 in the main manuscript.
(XLSX)

S5 Table Meta analysis performance metrics for neural network models. Meta-analysis of ILI rate forecasts around the peak of a flu season for FF, SRNN, and IRNN. *δ*-p denotes the temporal difference (in days) in forecasting the peak of the flu seasons 2015/16, 2016/17, 2017/18, and 2018/19, respectively. Negative / positive values indicate an earlier / later forecast. Avg. *δ*-*y*_p_ measures the average magnitude difference in the estimate of the peak of the flu season between a forecasting model and CDC. MAE-p is the MAE when the ILI rate is above the seasonal mean plus one standard deviation. SMAPE-p (%) is the symmetric mean absolute percentage of error for the same time periods.
(XLSX)

## References

[1] Ferguson NM, Laydon D, Nedjati-Gilani G, Imai N, Ainslie K, Baguelin M, et al. Impact of non-pharmaceutical interventions (NPIs) to reduce COVID-19 mortality and healthcare demand. Imperial College COVID-19 Response Team London. 2020;.

[2] Birrell P, Blake J, Van Leeuwen E, Gent N, De Angelis D. Real-time nowcasting and forecasting of COVID-19 dynamics in England: the first wave. Philos Trans R Soc B. 2021;376(1829):20200279. doi: 10.1098/rstb.2020.0279 34053254 PMC8165585

[3] Ioannidis JPA, Cripps S, Tanner MA. Forecasting for COVID-19 has failed. Int J Forecast. 2022;38(2):423–438. doi: 10.1016/j.ijforecast.2020.08.004 32863495 PMC7447267

[4] Shaman J, Karspeck A. Forecasting seasonal outbreaks of influenza. PNAS. 2012;109(50):20425–20430. doi: 10.1073/pnas.1208772109 23184969 PMC3528592

[5] Nsoesie E, Mararthe M, Brownstein J. Forecasting peaks of seasonal influenza epidemics. PLoS Curr. 2013;5. doi: 10.1371/currents.outbreaks.bb1e879a23137022ea79a8c508b030bc 23873050 PMC3712489

[6] Dugas AF, Jalalpour M, Gel Y, Levin S, Torcaso F, Igusa T, et al. Influenza Forecasting with Google Flu Trends. PLoS ONE. 2013;8(2):e56176. doi: 10.1371/journal.pone.0056176 23457520 PMC3572967

[7] Brooks LC, Farrow DC, Hyun S, Tibshirani RJ, Rosenfeld R. Flexible modeling of epidemics with an empirical Bayes framework. PLoS Comput Biol. 2015;11(8). doi: 10.1371/journal.pcbi.1004382 26317693 PMC4552841

[8] Zhang Q, Gioannini C, Paolotti D, Perra N, Perrotta D, Quaggiotto M, et al. Social Data Mining and Seasonal Influenza Forecasts: The FluOutlook Platform. In: Proc. of ECML PKDD; 2015. p. 237–240.

[9] Brownstein JS, Chu S, Marathe A, Marathe MV, Nguyen AT, Paolotti D, et al. Combining Participatory Influenza Surveillance with Modeling and Forecasting: Three Alternative Approaches. JMIR Public Health Surveill. 2017;3(4):e7344. doi: 10.2196/publichealth.7344 29092812 PMC5688248

[10] Zhang Q, Perra N, Perrotta D, Tizzoni M, Paolotti D, Vespignani A. Forecasting seasonal influenza fusing digital indicators and a mechanistic disease model. In: WWW; 2017. p. 311–319.

[11] Brooks LC, Farrow DC, Hyun S, Tibshirani RJ, Rosenfeld R. Nonmechanistic forecasts of seasonal influenza with iterative one-week-ahead distributions. PLoS Comput Biol. 2018;14(6). doi: 10.1371/journal.pcbi.1006134 29906286 PMC6034894

[12] Ray EL, Reich NG. Prediction of infectious disease epidemics via weighted density ensembles. PLoS Comput Biol. 2018;14(2). doi: 10.1371/journal.pcbi.1005910 29462167 PMC5834190

[13] Pei S, Kandula S, Yang W, Shaman J. Forecasting the spatial transmission of influenza in the United States. PNAS. 2018;115(11):2752–2757. doi: 10.1073/pnas.1708856115 29483256 PMC5856508

[14] Adhikari B, Xu X, Ramakrishnan N, Prakash BA. EpiDeep: Exploiting Embeddings for Epidemic Forecasting. In: Proc. of ACM SIGKDD; 2019. p. 577–586.

[15] Reich NG, Brooks LC, Fox SJ, Kandula S, McGowan CJ, Moore E, et al. A collaborative multiyear, multimodel assessment of seasonal influenza forecasting in the United States. PNAS. 2019;116(8):3146–3154. doi: 10.1073/pnas.1812594116 30647115 PMC6386665

[16] Lutz CS, Huynh MP, Schroeder M, Anyatonwu S, Dahlgren FS, Danyluk G, et al. Applying infectious disease forecasting to public health: a path forward using influenza forecasting examples. BMC Public Health. 2019;19(1). doi: 10.1186/s12889-019-7966-8 31823751 PMC6902553

[17] Reich NG, McGowan CJ, Yamana TK, Tushar A, Ray EL, Osthus D, et al. Accuracy of real-time multi-model ensemble forecasts for seasonal influenza in the U.S. PLoS Comput Biol. 2019;15(11). doi: 10.1371/journal.pcbi.1007486 31756193 PMC6897420

[18] Zimmer C, Yaesoubi R. Influenza forecasting framework based on Gaussian Processes. In: Proc. of ICML; 2020. p. 11671–11679.

[19] Miliou I, Xiong X, Rinzivillo S, Zhang Q, Rossetti G, Giannotti F, et al. Predicting seasonal influenza using supermarket retail records. PLoS Comput Biol. 2021;17(7):e1009087. doi: 10.1371/journal.pcbi.1009087 34252075 PMC8297944

[20] Venkatramanan S, Sadilek A, Fadikar A, Barrett CL, Biggerstaff M, Chen J, et al. Forecasting influenza activity using machine-learned mobility map. Nat Commun. 2021;12(1). doi: 10.1038/s41467-021-21018-5 33563980 PMC7873234

[21] Osthus D, Moran KR. Multiscale influenza forecasting. Nat Commun. 2021;12 (2991). doi: 10.1038/s41467-021-23234-5 34016992 PMC8137955

[22] Wagner M, Lampos V, Cox IJ, Pebody R. The added value of online user-generated content in traditional methods for influenza surveillance. Sci Rep. 2018;8(13963). doi: 10.1038/s41598-018-32029-6 30228285 PMC6143510

[23] Biggerstaff M, Alper D, Dredze M, Fox S, Fung ICH, Hickmann KS, et al. Results from the centers for disease control and prevention’s predict the 2013–2014 Influenza Season Challenge. BMC Infect Dis. 2016;16(357). doi: 10.1186/s12879-016-1669-x 27449080 PMC4957319

[24] McGowan CJ, Biggerstaff M, Johansson M, Apfeldorf KM, Ben-Nun M, Brooks L, et al. Collaborative efforts to forecast seasonal influenza in the United States, 2015–2016. Sci Rep. 2019;9(683). doi: 10.1038/s41598-018-36361-9 30679458 PMC6346105

[25] Biggerstaff M, Johansson M, Alper D, Brooks LC, Chakraborty P, Farrow DC, et al. Results from the second year of a collaborative effort to forecast influenza seasons in the United States. Epidemics. 2018;24:26–33. doi: 10.1016/j.epidem.2018.02.003 29506911 PMC6108951

[26] Marquez L, Hill T, O’Connor M, Remus W. Neural network models for forecast: a review. In: HICSS; 1992. p. 494–498.

[27] Adya M, Collopy F. How effective are neural networks at forecasting and prediction? A review and evaluation. J Forecast. 1998;17(5-6):481–495. doi: 10.1002/(SICI)1099-131X(1998090)17:5/6<481::AID-FOR709>3.0.CO;2-Q

[28] Jiang W, Luo J. Graph neural network for traffic forecasting: A survey. Expert Syst Appl. 2022;207(117921).

[29] Aiken EL, Nguyen AT, Viboud C, Santillana M. Toward the use of neural networks for influenza prediction at multiple spatial resolutions. Science Advances. 2021;7(25):eabb1237. doi: 10.1126/sciadv.abb1237 34134985 PMC8208709

[30] Kiureghian AD, Ditlevsen O. Aleatory or epistemic? Does it matter? Struct Saf. 2009;31(2):105–112. doi: 10.1016/j.strusafe.2008.06.020

[31] Kendall A, Gal Y. What Uncertainties Do We Need in Bayesian Deep Learning for Computer Vision? In: NeurIPS; 2017. p. 5580–5590.

[32] Ginsberg J, Mohebbi MH, Patel RS, Brammer L, Smolinski MS, Brilliant L. Detecting influenza epidemics using search engine query data. Nature. 2009;457(7232):1012–1014. doi: 10.1038/nature07634 19020500

[33] Lampos V, Cristianini N. Tracking the flu pandemic by monitoring the Social Web. In: CIP; 2010. p. 411–416.

[34] Lampos V, Miller AC, Crossan S, Stefansen C. Advances in nowcasting influenza-like illness rates using search query logs. Sci Rep. 2015;5:12760. doi: 10.1038/srep12760 26234783 PMC4522652

[35] Yang S, Santillana M, Kou SC. Accurate estimation of influenza epidemics using Google search data via ARGO. PNAS. 2015;112(47):14473–14478. doi: 10.1073/pnas.1515373112 26553980 PMC4664296

[36] Lampos V, Zou B, Cox IJ. Enhancing feature selection using word embeddings: The case of flu surveillance. In: WWW; 2017. p. 695–704.

[37] Zou H, Hastie T. Regularization and variable selection via the elastic net. Journal of the royal statistical society: series B (statistical methodology). 2005;67(2):301–320. doi: 10.1111/j.1467-9868.2005.00503.x

[38] Rasmussen CE, Williams CKI. Gaussian Processes for Machine Learning. MIT Press; 2006.

[39] Zou B, Lampos V, Cox IJ. Multi-Task Learning Improves Disease Models from Web Search. In: WWW; 2018. p. 87–96.

[40] Paul MJ, Dredze M, Broniatowski D. Twitter improves influenza forecasting. PLoS Curr. 2014;6. doi: 10.1371/currents.outbreaks.90b9ed0f59bae4ccaa683a39865d9117 25642377 PMC4234396

[41] Osthus D. Fast and accurate influenza forecasting in the United States with Inferno. PLoS Comput Biol. 2022;18(1). doi: 10.1371/journal.pcbi.1008651 35100253 PMC8830797

[42] Ray EL, Sakrejda K, Lauer SA, Johansson MA, Reich NG. Infectious disease prediction with kernel conditional density estimation. Stat Med. 2017;36(30):4908–4929. doi: 10.1002/sim.7488 28905403 PMC5771499

[43] Alom MZ, Taha TM, Yakopcic C, Westberg S, Sidike P, Nasrin MS, et al. A State-of-the-Art Survey on Deep Learning Theory and Architectures. Electronics. 2019;8(3). doi: 10.3390/electronics8030292

[44] Hernández-Lobato JM, Adams R. Probabilistic backpropagation for scalable learning of bayesian neural networks. In: ICML; 2015. p. 1861–1869.

[45] Yang W, Lipsitch M, Shaman J. Inference of seasonal and pandemic influenza transmission dynamics. PNAS. 2015;112(9):2723–2728. doi: 10.1073/pnas.1415012112 25730851 PMC4352784

[46] Baltrusaitis K, Noddin K, Nguyen C, Crawley A, Brownstein JS, White LF. Evaluation of approaches that adjust for biases in participatory surveillance systems. Online J Public Health Inform. 2018;10(1). doi: 10.5210/ojphi.v10i1.8908

[47] UK Health Security Agency. Weekly national Influenza and COVID-19 surveillance reports. Official Statistics (UKHSA). 2023;.

[48] Clemente L, Lu F, Santillana M, et al. Improved real-time influenza surveillance: using internet search data in eight Latin American countries. JPHS. 2019;5(2):e12214. doi: 10.2196/12214 30946017 PMC6470460

[49] Zou B, Lampos V, Cox IJ. Transfer Learning for Unsupervised Influenza-like Illness Models from Online Search Data. In: WWW; 2019. p. 2505–2516.

[50] Ning S, Yang S, Kou S. Accurate regional influenza epidemics tracking using Internet search data. Sci Rep. 2019;9 (5238). doi: 10.1038/s41598-019-41559-6 30918276 PMC6437143

[51] U.S. influenza surveillance: Purpose and methods; 2022. Available from: https://www.cdc.gov/flu/weekly/overview.htm.

[52] Lampos V, Majumder MS, Yom-Tov E, Edelstein M, Moura S, Hamada Y, et al. Tracking COVID-19 using online search. npj Digit Med. 2021;4(17). doi: 10.1038/s41746-021-00384-w 33558607 PMC7870878

[53] Gal Y. Uncertainty in deep learning. University of Cambridge. 2016;.

[54] Bishop CM. Mixture density networks. Aston University; 1994.

[55] Blei DM, Kucukelbir A, McAuliffe JD. Variational Inference: A Review for Statisticians. JASA. 2017;112(518):859–877. doi: 10.1080/01621459.2017.1285773

[56] Sharma M, Farquhar S, Nalisnick E, Rainforth T. Do Bayesian Neural Networks Need To Be Fully Stochastic? arXiv 221106291 (Preprint). 2022;.

[57] Honkela A, Raiko T, Kuusela M, Tornio M, Karhunen J. Approximate Riemannian conjugate gradient learning for fixed-form variational Bayes. JMLR. 2010;11:3235–3268.

[58] Jospin LV, Laga H, Boussaid F, Buntine W, Bennamoun M. Hands-On Bayesian Neural Networks—A Tutorial for Deep Learning Users. IEEE CIM. 2022;17(2):29–48.

[59] Graves A. Practical Variational Inference for Neural Networks. In: NeurIPS. vol. 24; 2011.

[60] Fu H, Li C, Liu X, Gao J, Celikyilmaz A, Carin L. Cyclical Annealing Schedule: A Simple Approach to Mitigating KL Vanishing. In: NAACL; 2019. p. 240–250.

[61] Shahriari B, Swersky K, Wang Z, Adams RP, De Freitas N. Taking the human out of the loop: A review of Bayesian optimization. Proc IEEE. 2015;104(1):148–175. doi: 10.1109/JPROC.2015.2494218

