# Supplementary material for: Neural network models for influenza forecasting with associated uncertainty using Web search activity trends
Source: PLoS Comput Biol. 2023 Aug 28;19(8):e1011392. doi: 10.1371/journal.pcbi.1011392 (PMC10491400; doi:10.1371/journal.pcbi.1011392)
Supplement: S1 Appendix — Appendix discussing the multibin logarithm score, forecast skill score, continuous ranked probability score, mean absolute error and bivariate correlation metric. As well as the persistence model used the baseline. (PDF) [file pcbi.1011392.s001.pdf]

S1 Appendix

Multibin logarithm score and forecast Skill score

The CDC use forecast Skill as a metric to compare forecasting models. For a forecast estimate $\hat{y} \in [0, 1]$, they define an 'accuracy of practical significance' as being within $\pm 0.5\%$ of the correct ILI rate $y \in [0, 1]$. The sum of the probability assigned to this region defines the Skill which is given by:

$$\text{Skill}\,(\hat{y}, y) = \left(\sum_{i=-5}^{5} p(y + i \times 0.01|\hat{y}) \right) , \quad (1)$$

where $p(y + i|\hat{y})$ is the probability assigned to a bin of size 0.1 around the true ILI rate y. To compute the Skill score for a normal distribution $\hat{\mathcal{N}} = \mathcal{N}(\hat{y}, \hat{\sigma})$, we first obtain the lower value of the correct ILI bin, i.e. $y_b = 0.1 \times \text{floor}(y \times 10)$, and then use the cumulative density function (`cdf`) of $\hat{\mathcal{N}}$ to compute:

$$\text{Skill}(\texttt{cdf}, y_b) = (\texttt{cdf}(y_b + 0.6) - \texttt{cdf}(y_b - 0.5)) \, . \quad (2)$$

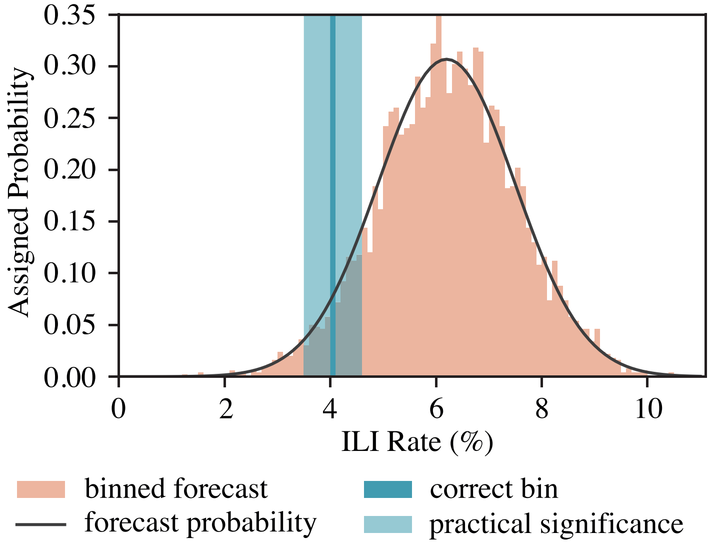

Fig A in S1 Appendix. Illustration of a binned probabilistic forecast, showing the correct bin and area of practical significance.

An example of a forecast and the binned ILI rate is given in Fig A in S1 Appendix. The true ILI rate y is equal to 4.03%, so the correct bin is between 4.0% and 4.1%. The area within ± 0.5 percentage points of the correct bin is considered an 'accuracy of practical significance' [1]. That is, from the bin between 3.5 and 3.6 up to 4.5 and 4.6. The probabilities assigned to these bins are: 0.0047, 0.0046, 0.0076, 0.0080, 0.01074, 0.0051, 0.0092, 0.0033, 0.0081, and 0.0060. The Skill score is simply the sum of these probabilities, i.e. 0.0673. When calculating the Skill score for several forecasts the geometric average is taken. For example, if for weeks 1 to 4 of a flu season we have forecast Skills of 0.53, 0.61, 0.40, 0.45, the skill for the four week period is equal to

$$\sqrt[4]{0.53 \times 0.61 \times 0.40 \times 0.45} = 0.49 \, . \quad (3)$$

When computing the average Skill for multiple seasons or forecast horizons the same geometric average is used.

The Skill score is not a strictly proper metric [2], i.e. it does not have a single unique ideal solution. This has been a source of criticism [3]. For example, with mean absolute error (MAE) there is only one estimate which will result in a 0 score. With forecast Skill, any forecast which places 100% probability within the area of practical significance will achieve a Skill score of 1.

Continuous ranked probability score

Contrary to forecast Skill, both negative log likelihood (NLL) and continuous ranked probability score (CRPS) are strictly proper [2]. A criticism of NLL is that it over-penalises errors where the difference between the actual and forecasted value is much greater than the associated uncertainty [2]. CRPS is more forgiving. It is defined by

$$\mathrm{CRPS}(\mathbf{y}, \hat{\mathbf{y}}, \hat{\sigma}) = \frac{1}{T}\sum_{t=1}^{T} \hat{\sigma}\left[\frac{1}{\sqrt{\pi}} - 2\varphi_t\left(\frac{y_t - \hat{y}_t}{\hat{\sigma}_t}\right) - \frac{y_t - \hat{y}_t}{\hat{\sigma}_t}\left(2\Psi_t\left(\frac{y_t - \hat{y}_t}{\hat{\sigma}_t}\right) - 1\right)\right], \quad (4)$$

where φ_t and Ψ_t respectively denote the probability density function and the cumulative distribution function of a standard Gaussian variable $\mathcal{N}(\hat{y}_t, \hat{\sigma}_t)$. CRPS is a probabilistic metric that generalises to MAE when the standard deviation is 0.

Both CRPS and NLL favour a confident and accurate forecast. CRPS, however, is more forgiving when the confidence is high and the accuracy is poor. Fig B in S1 Appendix illustrates this point. Here the blue and green curves depict the NLL and CRPS scores, respectively. Estimates are represented by the red diagonal line. The true value to be predicted is $y = 0$. The first point on the diagonal line has zero standard deviation, but predicts $y = -1$, i.e. an erroneous value with perfect confidence (zero uncertainty). The CRPS penalises this with a score of 1 (which in this case is equal to the MAE). In contrast, the NLL tends to infinity. As we move from left to right, the error in y is initially decreasing while our uncertainty is increasing. As our estimate approaches the true value of $y = 0$ which occurs when the standard deviation (x-axis) is ≈ 0.34, both curves approach a minimum value. We would like to note that the minimum value of NLL is closer to when $y = 0$, but the minimum of CRPS happens prior to that (i.e. around point 0.3 on the x-axis). As we continue to move from left to right, the error in y increases along with the uncertainty. At the right-most side, we have $y = 0.5$ with a standard deviation of 0.5. Here, the CRPS score is approximately 0.2, and the NLL 0.7. Overall, the NLL metric much more strongly penalises errors that are outside of the uncertainty region. We note this, but do not favour one metric over the other, reporting both in our results.

Mean absolute error and bivariate correlation

We report the mean absolute error (MAE) and bivariate correlation (r). We measure MAE and r between the means of the forecasted estimates ($\hat{\mathbf{y}} \in \mathbb{R}^T_{[0,1]}$) and the ground truth ILI rates ($\mathbf{y} \in \mathbb{R}^T_{[0,1]}$) during a flu season. The MAE evaluates how close the forecasted values are to the true values

$$\mathrm{MAE} = \frac{1}{T}\sum_{t=1}^{T} |\hat{y}_t - y_t| \, . \quad (5)$$

The bivariate correlation r evaluates how similar the shape of the forecasted flu season is to the ground truth

$$r = \frac{\sum_{t=1}^{T} (\hat{y}_t - \bar{\hat{y}})(y_t - \bar{y})}{\sqrt{\sum_{t=1}^{T} (\hat{y}_t - \bar{\hat{y}})^2 \sum_{t=1}^{T} (y_t - \bar{y})^2}} \, . \quad (6)$$

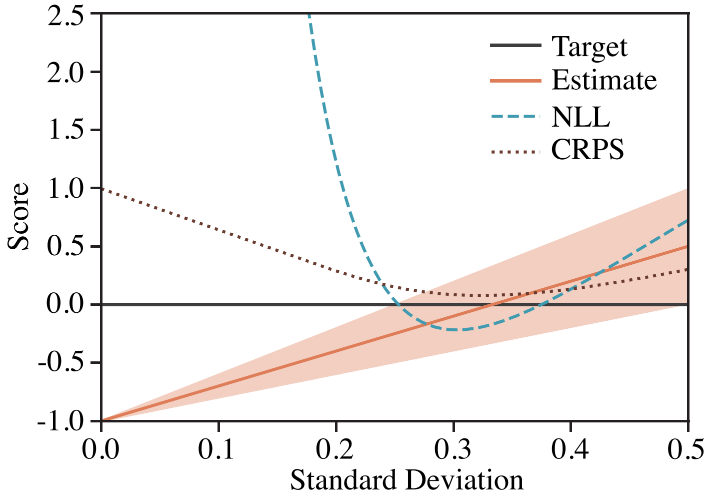

Fig B in S1 Appendix. NLL and CRPS variation with changing error and confidence. The red line shows a model's estimate with standard deviation shown in pink where the true value is $y = 0$. As the accuracy and confidence change, the CRPS and NLL values have different trajectories.

Persistence model

A simple persistence model (PER) uses the last available ground truth value to make a forecast. For example, assume that the last observed ILI rate at time point t_0 is equal to y_0. In this case, at time point t_0 the n-day ahead forecast of a persistence model will always be equal to y_0, i.e. $\hat{y}_{t_0+n} = y_0$.

References

1. Reich NG, Brooks LC, Fox SJ, Kandula S, McGowan CJ, Moore E, et al. A collaborative multiyear, multimodel assessment of seasonal influenza forecasting in the United States. PNAS. 2019;116(8):3146–3154.

2. Gneiting T, Raftery AE. Strictly proper scoring rules, prediction, and estimation. JASA. 2007;102(477):359–378.

3. Bracher J. On the multibin logarithmic score used in the FluSight competitions. PNAS. 2019;116(42):20809–20810.
